# Phase I study of pemetrexed with sorafenib in advanced solid tumors

**DOI:** 10.18632/oncotarget.9434

**Published:** 2016-05-18

**Authors:** Andrew Poklepovic, Sarah Gordon, Danielle A. Shafer, John D. Roberts, Prithviraj Bose, Charles E. Geyer, William P. McGuire, Mary Beth Tombes, Ellen Shrader, Katie Strickler, Maria Quigley, Wen Wan, Maciej Kmieciak, H. Davis Massey, Laurence Booth, Richard G. Moran, Paul Dent

**Affiliations:** ^1^ Department of Massey Cancer Center, Virginia Commonwealth University, Richmond, Virginia, USA; ^2^ Department of Internal Medicine, Virginia Commonwealth University, Richmond, Virginia, USA; ^3^ Department of Biostatistics, Virginia Commonwealth University, Richmond, Virginia, USA; ^4^ Department of Pathology, Virginia Commonwealth University, Richmond, Virginia, USA; ^5^ Department of Biochemistry and Molecular Biology, Virginia Commonwealth University, Richmond, Virginia, USA; ^6^ Department of Pharmacology and Toxicology, Virginia Commonwealth University, Richmond, Virginia, USA; ^7^ Current address: Department of Medical Oncology, Yale School of Medicine, New Haven, Connecticut, USA; ^8^ Current address: Department of Leukemia, The University of Texas MD Anderson Cancer Center, Houston, Texas, USA

**Keywords:** clinical trial, pemetrexed, solid tumors, sorafenib

## Abstract

**Purpose:**

To determine if combination treatment with pemetrexed and sorafenib is safe and tolerable in patients with advanced solid tumors.

**Results:**

Thirty-seven patients were enrolled and 36 patients were treated (24 in cohort A; 12 in cohort B). The cohort A dose schedule resulted in problematic cumulative toxicity, while the cohort B dose schedule was found to be more tolerable. The maximum tolerated dose (MTD) was pemetrexed 750 mg/m^2^ every 14 days with oral sorafenib 400 mg given twice daily on days 1–5. Because dosing delays and modifications were associated with the MTD, the recommended phase II dose was declared to be pemetrexed 500 mg/m^2^ every 14 days with oral sorafenib 400 mg given twice daily on days 1–5. Thirty-three patients were evaluated for antitumor activity. One complete response and 4 partial responses were observed (15% overall response rate). Stable disease was seen in 15 patients (45%). Four patients had a continued response at 6 months, including 2 of 5 patients with triple-negative breast cancer.

**Experimental Design:**

A phase I trial employing a standard 3 + 3 design was conducted in patients with advanced solid tumors. Cohort A involved a novel dose escalation schema exploring doses of pemetrexed every 14 days with continuous sorafenib. Cohort B involved a modified schedule of sorafenib dosing on days 1–5 of each 14-day pemetrexed cycle. Radiographic assessments were conducted every 8 weeks.

**Conclusions:**

Pemetrexed and intermittent sorafenib therapy is a safe and tolerable combination for patients, with promising activity seen in patients with breast cancer.

## INTRODUCTION

Pemetrexed, an anti-folate chemotherapeutic agent, was developed as an inhibitor of thymidylate synthase and is approved for the treatment of advanced non-small cell lung cancer and mesothelioma. It has also demonstrated modest activity in previously untreated breast cancer [1]. Pemetrexed has been shown to have more than one mechanism of action in tumor cells as illustrated by the continued anti-proliferative effect of the drug in cells exposed to exogenous thymidine (which prevents the cytotoxic effects of thymidylate synthase inhibition) [2–5]. A secondary target of pemetrexed has more recently been shown to be aminoimidazolecarboxamide ribonucleotide formyltransferase (AICART), the downstream folate-dependent enzyme in *de novo* purine synthesis [2, 3]. ZMP, the substrate of AICART, accumulates in pemetrexed-treated tumor cells, strongly suggesting that the reaction catalyzed by AICART is the step in purine synthesis that becomes rate-limiting after drug treatment [2]. Increasing concentrations of ZMP activate AMP-activated protein kinase (AMPK), which subsequently inhibits the mammalian target of rapamycin (mTOR). The activation of AMPK [2] and inactivation of mTOR [6] increases autophagosome formation. Pemetrexed also induces the formation of toxic acidic vesicular organelles, supporting the hypothesis that pemetrexed causes tumor cell death by autophagy [7].

Sorafenib is FDA-approved for the treatment of advanced hepatocellular carcinoma, renal cell carcinoma, and thyroid cancer. It is a multi-kinase inhibitor that was originally developed as an inhibitor of RAF-1, a component of the ERK1/2 pathway. Sorafenib was subsequently found to inhibit multiple other kinases including class III tyrosine kinase receptors such as platelet-derived growth factor receptor (PDGFR), vascular endothelial growth factor receptor (VEGFR), c-kit, and *fms*-like tyrosine kinase (FLT-3) [8–13]. Sorafenib also mediates down-regulation of MCL-1, a cytoprotective protein, through inhibition of protein translation, a process mediated by endoplasmic reticulum stress signaling [13, 14]. Reduced MCL-1 levels due to sorafenib exposure have been linked in many tumor types to increased levels of mitochondrial dysfunction and apoptosis [13, 15, 16]. Although high-dose sorafenib exposure (10 μM) increases the levels of autophagic markers, including increased numbers of LC3-GFP vesicles and elevated expression of Beclin 1 and ATG5, lower, more clinically relevant, sorafenib concentrations (~1–3 μM) were found to only cause a modest transient alteration in autophagy flux or expression of regulatory proteins [13, 15, 17, 18].

Because sorafenib and pemetrexed exert their effects through different pathways, we hypothesized that the drug combination would lead to a synergistic increase in autophagy and enhanced tumor cell killing. Pemetrexed and sorafenib were subsequently shown to interact in a greater than additive fashion to kill a wide variety of tumor cell types *in vitro* and in several models of mammary carcinoma *in vivo*, the latter without any apparent deleterious effects on normal tissues as judged by histologic staining or reduced animal body mass [6, 19]. In these prior studies, we demonstrated that pemetrexed and sorafenib interacted to kill tumor cells through endoplasmic reticulum stress signaling, inactivation of the PI3K/mTor pathway, and the induction of a toxic form of autophagic flux [6, 19]. Treatment of mammary carcinoma cells with pemetrexed and sorafenib reduced expression of the chaperone GRP78/BiP/HSPA5 in parallel with increased phosphorylation of eIF2α serine 51, all indicative of an endoplasmic reticulum stress response being induced by the drug combination [20]. Knock-down of PKR-like endoplasmic reticulum kinase (PERK) prevented the stimulation of eIF2α phosphorylation, in agreement with GRP78/BiP/HSPA5 being an inhibitor of PERK activity [20–22]. Pemetrexed and sorafenib increased the number of autophagosomes in cells as judged by an increase in the mean number of punctate LC3-GFP bodies per cell, an effect that was prevented by expression of dominant negative eIF2α S51A [20]. Over-expression of GRP78/BiP/HSPA5, or expression of dominant negative eIF2α S51A, significantly reduced the lethality of pemetrexed and sorafenib treatment [20]. Thus, by reducing chaperone expression and increasing endoplasmic reticulum stress signaling, treatment with the drug combination leads to a toxic form of autophagy.

Given the preclinical data, a single-center phase I clinical trial of the pemetrexed and sorafenib drug combination was initiated in patients with advanced solid tumor malignancies. A novel 3 + 3 dose-escalation design was used, with escalating doses of pemetrexed (500–1,000 mg/m^2^; IV) given every 14 days and sorafenib (200–400 mg; orally; twice daily) given continuously (cohort A). Dose-limiting toxicity (DLT) was assessed during the initial 4-week period. Because of unexpected and cumulative toxicity in cohort A, cohort B received a modified schedule of intermittent sorafenib dosing on days 1–5 of each pemetrexed administration (every 14 days). Radiographic response assessments were conducted approximately every 8 weeks using Response Evaluation Criteria for Solid Tumors (RECIST) v1.1.

## RESULTS

### Patient characteristics

Between October 2011 and December 2014, 37 patients were enrolled and 36 were treated. One patient withdrew prior to initiating study treatment. Twenty-four patients were treated in cohort A and 12 in cohort B. Thirty-three patients were evaluable for antitumor activity. This study was for all advanced solid tumors, but breast cancer was the most common tumor type enrolled (12 patients), including 5 with triple-negative disease and one male breast cancer patient. Baseline characteristics of the patients are summarized in Table 1.

**Table 1:** Characteristics of 36 patients treated

Characteristic	Number (%)
Age, y	
Median	59.5
Range	35–78
Gender	
Female	24 (67)
Male	12 (33)
Race	
Asian	1 (3)
Black or AA	11 (30)
White	24 (67)
Tumor type	
Adenoid cystic carcinoma	1 (3)
Breast, female, ER^−^, PR^−^, HER2^−^	5 (14)
Breast, female, ER^−^, PR^−^, HER2^+^	1 (3)
Breast, female, ER^+^, PR^−^, HER2^−^	1 (3)
Breast, female, ER^+^, PR^+^, HER2^+^	1 (3)
Breast, female, ER^+^, PR^+^, HER2^−^	2 (6)
Breast, female, ER^+^, PR^+^, HER2^+^	1 (3)
Breast, male	1 (3)
Cervix	2 (6)
Cholangiocarcinoma	1 (3)
Chondrosarcoma	1 (3)
Colon	3 (8)
Hepatocellular carcinoma	2 (6)
Kidney	1 (3)
Lung, adenocarcinoma	1 (3)
Lung, squamous cell carcinoma	1 (3)
Neuroendocrine carcinoma	1 (3)
Ovary	5 (14)
Pancreas	2 (6)
Soft tissue sarcoma	1 (3)
Thymus	1 (3)
Unknown primary, squamous cell carcinoma	1 (3)
Study treatment, weeks	
Mean	15.9
Median	10.2
Range	1–52

### Dose escalation and DLTs

In cohort A, a total of 6 DLT events occurred (Table 2). No DLTs were seen at dose levels A1 and A2. Two DLTs, both grade 3 mucositis, were seen at dose level A3. The dose escalation matrix used in cohort A indicated that the next dose level, A4, employ a reduced dose of pemetrexed with the dose of sorafenib maintained, given that the DLT seen at A3 was most likely pemetrexed-related. Therefore, pemetrexed dosing was reduced from 1,000 mg/m^2^ to 750 mg/m^2^ (dose level A4). Two DLTs, deemed to be pemetrexed-related, were observed at dose level A4 (grade 4 cytopenias [including leucopenia, neutropenia, lymphopenia, and thrombocytopenia] and grade 3 increases in ALT and AST). The next cohort (A5) employed a lower dose of pemetrexed (500 mg/m^2^) with sorafenib 400 mg given orally twice daily. Two DLTs (grade 4 hypertension and grade 4 hepatic failure) occurred at dose level A5. Enrollment resumed at the initial enrollment dose level A1 (pemetrexed 500 mg/m^2^ with sorafenib 200 mg given orally twice daily) and no DLTs were seen. Dose level A1 was defined as the initial MTD for cohort A: 500 mg/m^2^ pemetrexed IV every 14 days with 200 mg sorafenib orally twice daily, continuously. After the DLT period, however, delayed grade 3 toxicities including enterocolitis, nausea, vomiting, and dehydration were observed in one patient. Due to this and cumulative toxicities requiring dose modifications in cohort A, the protocol was revised and cohort A was closed to further accrual, and it was concluded that pemetrexed at any dose with continuous sorafenib was not tolerable. As the preclinical data supported concurrent drug administration, pulsatile dosing of sorafenib during pemetrexed exposure was therefore evaluated to maintain combination treatment exposure and to provide drug-free intervals. Cohort B was opened to explore intermittent dosing of sorafenib (days 1–5) with each dose of pemetrexed (IV every 14 days). Dose escalation continued to dose level B8 of 750 mg/m^2^ pemetrexed (IV every 14 days) with 400 mg sorafenib (orally twice a day, days 1–5). No DLTs occurred in cohort B. The MTD was not reached in cohort B and based upon the improved tolerance and response patterns with pemetrexed at 500 mg/m^2^, dose level B7 was declared to be the RP2D.

**Table 2:** Dose escalation and dose limiting toxicities (DLTs)

Dose Level	Pemetrexed (mg/m^2^)	Sorafenib (mg)	Patients, *n*	DLTs, *n*	DLT event	Weeks of treatment, median (range)
**Cohort A**	Every 14 days, IV	Continuous (D1–14), twice daily, oral				
A1	500	200	8	0		7.6 (2.6–51.0)
A2	750	400 in AM, 200 in PM	3	0		5.0 (4.9–16.0)
A3	1000	400	2	2	Grade 3 mucositis (2 patients)	24.1 (9.0–39.1)
**Cohort B**	Every 14 days, IV	Intermittent (D1–5), twice daily, oral				
B6	500	200	3	0		18.0 (16.0–52.0)
B7	500	400	3	0		19.6 (8.0–52.3)
B8	750	400	6	0		16.7 (9.0–26.9)

### Safety and tolerability

The median duration of treatment was 7 weeks for cohort A, 18 weeks for cohort B, and 10 weeks for all cohorts combined. Duration on treatment was longer in cohort B than cohort A due to both decreased toxicity and longer disease response. The median length of treatment for each dose level is shown in Table 2.

Common (occurring in at least 25% of patients) grade 2 and all grade 3 and 4 adverse events possibly, probably, or definitely related to study treatment are shown in Tables 3 and 4.

**Table 3:** Common (occurring in ≥ 25% of patients) grade 2 adverse events possibly, probably, definitely related to study treatment

Adverse event	# patients (% patients)			
	Cohort A		Cohort B	
	*n*= 24		*n* = 12	
	Grade 2		Grade 2	
Alkaline phosphatase increased	7	(29)		
Anemia	15	(63)	5	(42)
Anorexia			5	(42)
Fatigue	14	(58)	4	(33)
Lymphocyte count decreased	17	(71)	8	(67)
Malaise			5	(42)
Mucositis oral			3	(25)
Nausea			5	(42)
Neutrophil count decreased	9	(38)	7	(58)
Palmar-plantar erythrodysesthesia syndrome	10	(42)		
White blood cell decreased	9	(38)	7	(58)

**Table 4:** All grade 3 and 4 adverse events possibly, probably, definitely related to study treatment

Adverse event	# patients (% patients)							
	Cohort A				Cohort B			
	*n* = 24				*n* = 12			
	Grade 3		Grade 4		Grade 3		Grade 4	
Alanine aminotransferase increased	1	(4)						
Alkaline phosphatase increased	1	(4)						
Anemia	7	(29)			3	(25)		
Aspartate aminotransferase increased	1	(4)						
Blood bilirubin increased	1	(4)						
Cardiac troponin I increased	1	(4)						
Chest wall pain					1	(8)		
Dehydration	1	(4)			1	(8)		
Diarrhea	1	(4)						
Dyspnea					1	(8)		
Enterocolitis	1	(4)						
Fatigue	5	(21)			4	(33)		
Febrile neutropenia	2	(8)						
Generalized muscle weakness	1	(4)						
Hepatic failure			1	(4)				
Hyperkalemia			1	(4)				
Hypertension	7	(29)	1	(4)	1	(8)		
Hypocalcemia			1	(4)				
Hyponatremia	1	(4)						
Hypophosphatemia	2	(8)						
Lymphocyte count decreased	11	(46)	2	(8)	5	(42)		
Maculo-papular rash	1	(4)			1	(8)		
Mucositis oral	3	(13)						
Nausea	1	(4)						
Neutrophil count decreased	6	(25)	2	(8)	3	(25)		
Platelet count decreased	1	(4)	2	(8)	2	(17)	1	(8)
Sepsis			1	(4)				
Skin infection	1	(4)						
Upper respiratory infection	1	(4)						
Vomiting	1	(4)			1	(8)		
White blood cell decreased	9	(38)	3	(13)	5	(42)		

Grade 3 hypertension occurred more often in cohort A (29%) than cohort B (8%). There were no episodes of grade 3 oral mucositis in cohort B. Thirteen percent of patients in cohort A developed mucositis. Also, grade 2 palmar-plantar erythrodysesthesia (PPE, hand-foot syndrome) was seen in 42% of patients in cohort A and no patients in cohort B, clearly related to the change in sorafenib dosing from continuous to intermittent.

In general, fewer grade 4 adverse events were observed in cohort B compared to cohort A. One patient in cohort A, but none in cohort B, developed grade 4 hypertension. Other grade 4 adverse events observed in cohort A included the following: leucopenia (3 patients); lymphopenia, neutropenia, thrombocytopenia (each occurring in 2 patients); and hepatic failure, hyperkalemia, hypocalcemia and sepsis (each occurring in 1 patient). The grade 4 hepatic failure, with elevated ammonia, bilirubin, and alkaline phosphatase, occurred in the setting of hepato-renal syndrome due to massive and progressive intrahepatic metastasis from cholangiocarcinoma. The only grade 4 adverse event in cohort B was thrombocytopenia in 1 patient. The patient did not have any associated bleeding episodes.

Five treatment-unrelated deaths occurred on study, all in cohort A, with 3 occurring during treatment and 2 during follow-up. No study-related deaths occurred. One patient with a history of chronic aspiration experienced respiratory failure due to aspiration pneumonia, which was deemed unlikely related to study treatment. A patient with severe disease-related bowel obstruction died due to aspiration. A patient with rapid disease progression also developed hepato-renal syndrome with hyperkalemia, and it was felt the study drugs may have contributed to some of the decline in liver function. The patient declined further intervention and died due to myocardial infarction and not as a result of therapy. Two deaths due to disease progression occurred during follow-up and within 30 days after treatment was stopped for progressive disease.

One patient discontinued treatment due to adverse events. The patient (cohort A), with an ongoing objective PR, had breast cancer metastatic to the liver and developed liver function abnormalities (grade 3 bilirubin, grade 1 ALT, AST and alkaline phosphatase increases) in the twelfth month of study therapy. These abnormalities persisted despite stenting, which prohibited further protocol therapy.

Five patients withdrew from study treatment but remained in study follow-up. The first patient with hepatocellular cancer (cohort A) opted to discontinue study treatment following sequential sorafenib dose reductions for PPE that would have required continuation with pemetrexed alone. The second patient (cohort A) withdrew to transfer to hospice. The third patient (cohort A) experienced DLT (grade 4 hypertension) and withdrew from study declining recommended dose reductions. The fourth patient (cohort B), after 8 cycles of study treatment and a best response of stable disease, withdrew to pursue immunotherapy on another clinical trial. The fifth patient (cohort B) chose to withdraw due to a constellation of side effects from therapy including grade 3 fatigue, grade 2 malaise, and prolonged cytopenias (grade 2 neutropenia, grade 4 thrombocytopenia, grade 2 leucopenia, and grade 2 lymphopenia), making a decision to forego further recommended dose reductions.

Twenty-seven patients had study treatment discontinued due to disease progression.

### Disease response

**Figure 1:**
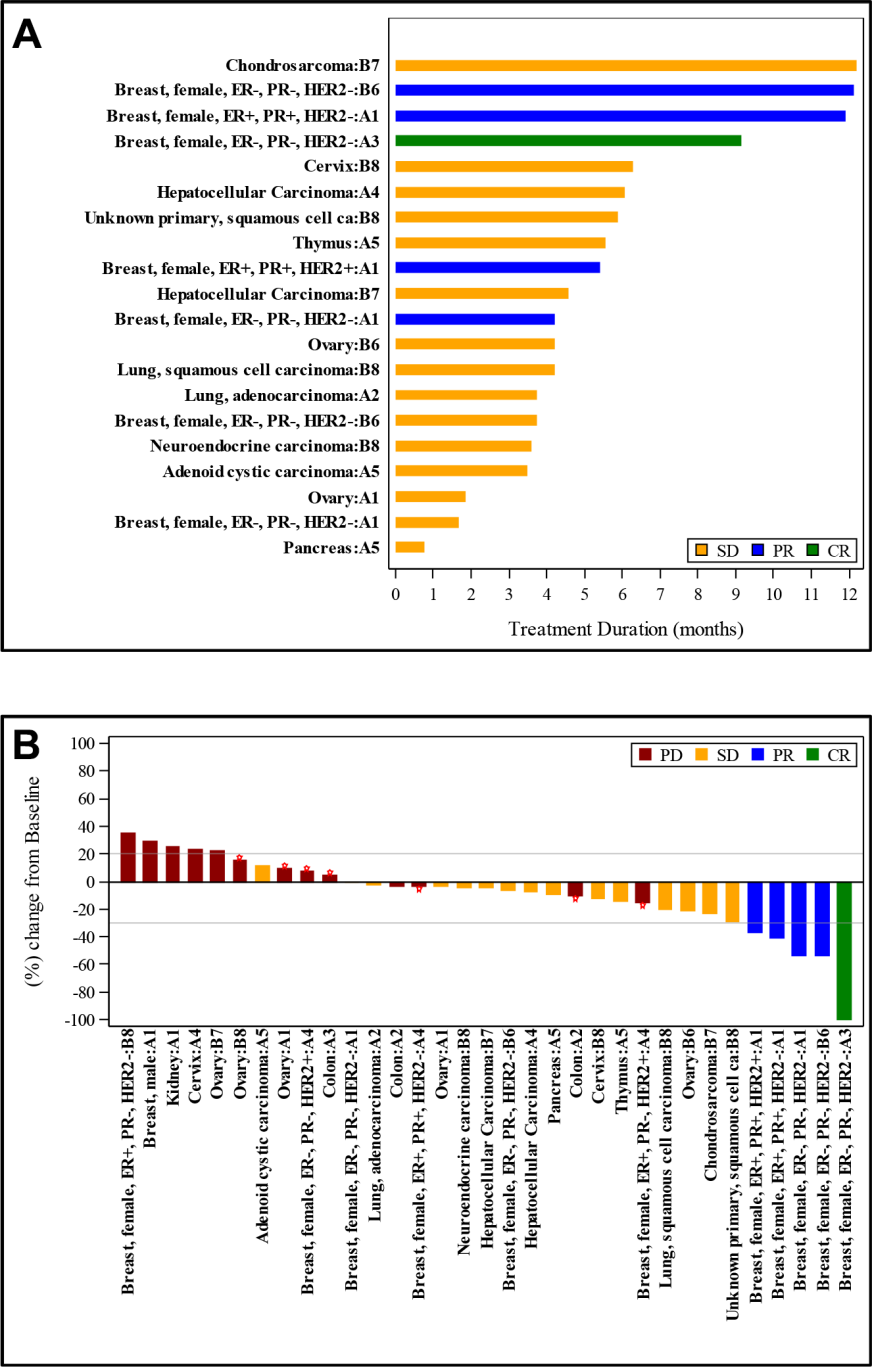
Treatment duration and tumor size change from baseline for patients with a best response of stable disease (SD), partial response (PR), or complete response (CR) (A), each bar represents the treatment duration of an individual patient. The tumor type and cohort for each patient is given on the y-axis. (**B**), each bar represents the maximum change in tumor size in comparison to baseline for an individual patient. The tumor type and cohort for each patient is given on the x-axis.

Although this study was not powered to assess response, 1 CR and 4 PRs were observed among the 33 response-evaluable patients for an overall response rate of 15%. SD was seen in 15 patients (45%). Best response to therapy is illustrated in a waterfall plot (Figure 1A). Long-term responses were seen, with 4 patients having continued responses at 6 months. Three of the 4 patients with continued responses had breast cancer, with the duration of best response ranging from 6 to 10 months. Two of those 3 patients had triple-negative breast cancer. The fourth patient had chondrosarcoma and despite a period of rapid growth of his tumor prior to enrollment onto study, remained on study with stable disease for 12 months. Nine patients (27%) with at least stable disease remained on study for 6 or more months. The duration of therapy for patients with a best response of SD or better is illustrated in a swimmers' plot (Figure 1B).

**Figure 2:**
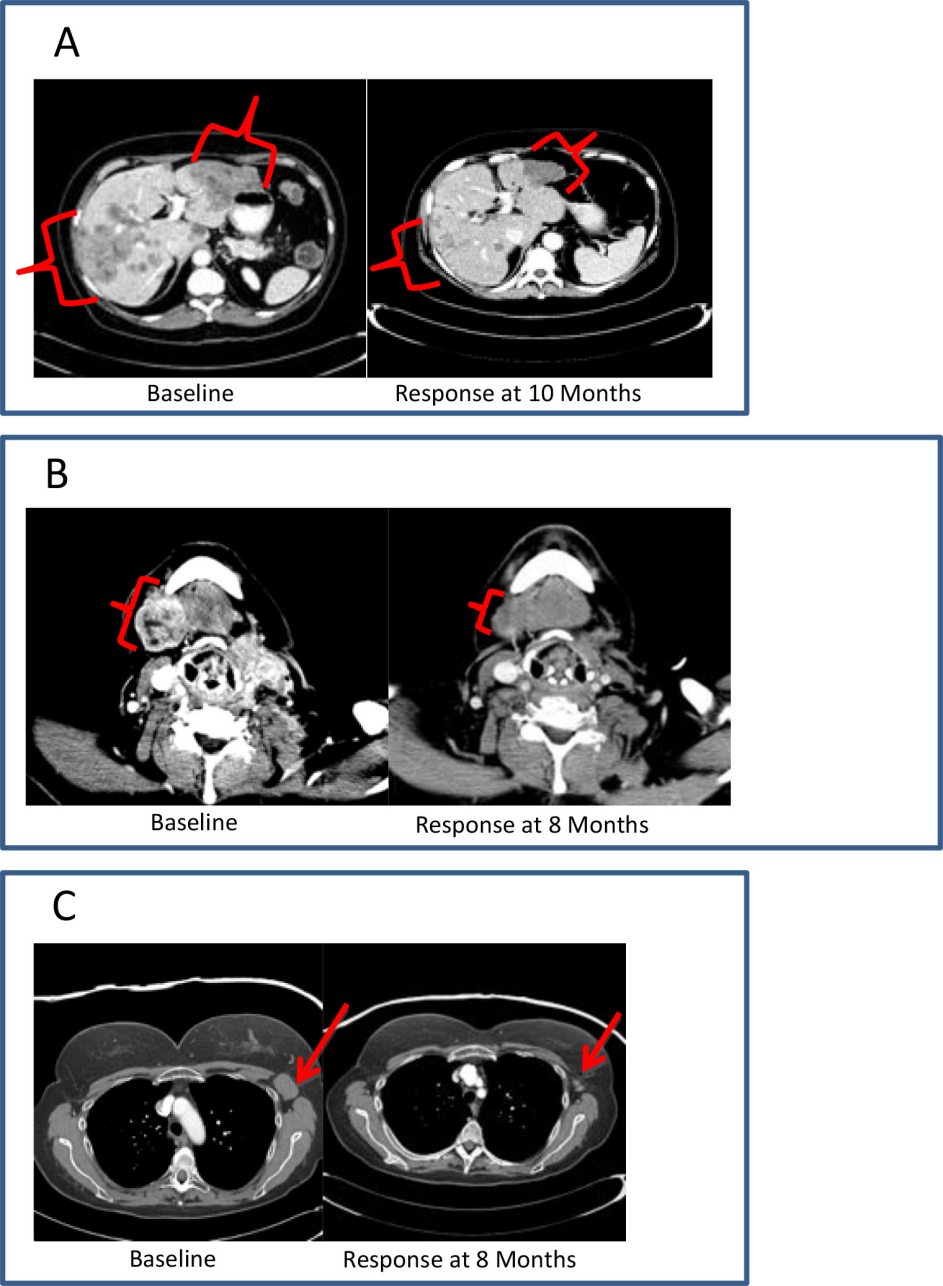
Clinical vignettes (**A**), diagnosis: hormone receptor positive breast cancer; previous treatment: 6 lines of systemic therapy; best response: PR; duration of therapy: 12 months. (**B**), diagnosis: initially ER+, Her-2- breast cancer, with subsequent development of triple-negative metastatic disease; previous treatment: 5 lines of systemic therapy; best response: PR; duration of therapy: 12 months. (**C**), diagnosis: triple-negative breast cancer; previous treatment: 7 lines of systemic therapy; best response: PR; duration of therapy: 9 months.

Among patients with breast cancer, cutaneous metastases, nodal metastases, and visceral disease were all observed to respond to therapy (Figure 2). Transient inflammatory responses were observed in a few patients before an objective response was observed.

### Biomarker studies

IHC staining was performed to determine the expression of Beclin 1, PTEN, and PDGFRβ in archived tumor samples from 18 of 33 patients who received treatment and were evaluable for response (Table 5). Samples were evaluated by a board certified pathologist, and scored for both intensity of the immunostain and percentage of cells staining positive. All samples stained positive for Beclin 1 and PDGFRβ. The intracellular location of Beclin 1 staining, cytoplasm versus perinuclear, was not associated with patient response. In contrast, samples from 5 patients did not express PTEN, and samples from another 3 patients expressed PTEN in less than 40% of the tumor cells. Of the 5 patients with no tumor expression of PTEN by IHC, there were 2 PRs (duration of therapy 18 and 52 weeks) and one durable SD (chondrosarcoma, duration of therapy 52 weeks). All 3 patients with low PTEN expression (< 40% of tumor cells positive and an intensity of “+” by IHC) had SD (duration of therapy 16, 18, and 25 weeks). In contrast, among the 10 patients with high expression (≥ 50%), 3 had SD, and none had a PR. The ordinal regression results indicate a significant association between no or low expression of PTEN and the ordinal clinical response (*p* = 0.028). Excluded from the above analysis was a patient who did not have PTEN staining done as part of the study, but who did have prior PTEN evaluation done by commercial assay (CARIS Life Sciences), the results of which showed low PTEN expression. This patient had a complete response (CR, duration of therapy of 39 weeks), which if included in the above analysis, would result in stronger correlations between the expression levels of PTEN and the response (*p* = 0.0131).

**Table 5:** Association between PTEN expression and patient response

Patient	PTEN^a^		Best response^b^	Duration on therapy (weeks)
	%	Intensity		
1	0	0	PR	18
2	0	0	PD	6
3	0	0	PR	52
4	0	0	SD	52
5	0	0	PD	11
6^c^	15	+	CR	39
7	20	+	SD	18
8	20	+	SD	25
9	35	+	SD	16
10	50	+	PD	3
11	60	+	SD	27
12	70	+	PD	5
13	90	++	SD	15
14	95	+	PD	7
15	100	++	PD	9
16	100	++	PD	10
17	100	++	PD	7
18	100	++	PD	9
19	100	++	SD	15

a Archival tumor tissue was analyzed by immunohistochemistry for percentage of tumor cells staining positive for PTEN expression and for the intensity of staining (0, +, ++, +++). Intensity of staining was determined by a board certified pathologist using commonly used methods of assessing IHC stain intensity.

b Best response was described as one of the following: CR: complete response; PD: progressive disease; PR: partial response; SD: stable disease.

c The evaluation of PTEN expression for this patient was done by a commercial assay (CARIS Life Sciences) prior to enrollment in the study.

## DISCUSSION

This phase I drug combination study evaluated the safety of the combination of pemetrexed with sorafenib for the treatment of patients with advanced solid tumors. The study was predicated on the hypothesis that pemetrexed and sorafenib would kill cancer cells and reduce tumor growth via a toxic form of autophagy. The initial study design (cohort A) utilized the standard dosing of sorafenib (twice daily continuously) along with pemetrexed. DLT events occurred at each dose level in cohort A when the dose of sorafenib was escalated to 400 mg twice daily continuously, regardless of the dose of pemetrexed (Table 2). All patients in cohort A had appropriate organ function and vitamin B12 and folate administration at the time of study entry, and no clear additional risk factors or predictors of enhanced toxicity could be identified in the patients who experienced DLT. Due to this, methylmalonic acid and homocysteine levels were monitored prior to enrollment in cohort B. However, with no DLTs seen in cohort B, no conclusions could be drawn about interactions between those levels and risk of DLT.

Additionally, a cumulative, sorafenib-mediated toxicity developed after the DLT observation period in patients on cohort A and led to sorafenib dose modifications for the remainder of therapy. In one patient on dose level A1, the first 2 doses of combination therapy were well tolerated; however, the third dose led to profound and long-lasting cytopenias, as well as enteric and hepatic injury. Following the development of substantial toxicity in this patient outside of the standard DLT evaluation period, the decision was made to halt accrual to cohort A (continuous sorafenib dosing) and revise the protocol to define and initiate accrual to cohort B (intermittent sorafenib dosing). It is hypothesized that continuous administration of sorafenib may have primed some patients for enhanced hematologic and non-hematologic toxicity from pemetrexed administration, while other patients were able to continue therapy with continuous sorafenib extended exposure to the drug combination. Reasons for this discrepant outcome remain unclear after an extensive search for pre-entry differences between the two groups.

Given the experience in cohort A, the decision was made to investigate intermittent dosing of sorafenib, to allow dose escalation of the combination, with inclusion of drug-free intervals for patient recovery, based on pre-clinical data. The study was reopened with cohort B, utilizing a standard 3 + 3 dose escalation design, with pemetrexed given once every 14 days and sorafenib daily for 5 days starting with each pemetrexed dose. This intermittent dosing of sorafenib in cohort B reduced the toxicity seen with the drug combination, and all pre-planned dose levels were explored without any DLTs identified (Table 2). Generally, treatment in cohort B was very well tolerated. Of the 12 patients treated in cohort B, 1 patient at the highest dose of pemetrexed (750 mg/m^2^) developed reversible but prolonged toxicity in the fourth month (grade 3 fatigue, grade 3 anemia, grade 4 thrombocytopenia). The patient declined further dose reduction and opted to withdraw from study treatment. No subjects in cohort B were removed from the study due to adverse events.

One of the 3 patients treated at dose level B7 (pemetrexed 500 mg/m^2^, sorafenib 400 mg^2^ twice daily) required a dose delay and was treated every 21 days instead of every 14 days. This patient tolerated treatment well after increasing the interval between pemetrexed doses. At dose level B8 (pemetrexed 750 mg/m^2^, sorafenib 400 mg twice daily), 4 of the 6 patients treated required a dose delay. Two of these patients also required a dose reduction.

Patients in both cohorts were observed to have remarkably durable responses as well as SD, especially given the heavy pretreatment that is typical in this population. Forty-five percent of patients had SD and an average length on treatment of 19 weeks. Seventeen patients were treated for 3 or more months, including 75% of patients on cohort B. Sustained treatment responses lasting six months or more were seen in 4 patients and response duration ranged from 6 to 10 months. PR or SD was seen in 75% of patients on cohort B.

Enrollment of 12 patients with breast cancer on this trial presented an opportunity to gain more information about this therapy in this disease population. An objective response or SD was observed in 58% of breast cancer patients (7 out of 12 patients). Three patients with breast cancer had sustained responses lasting 6 months or more and remained on treatment for 9 to 12 months. All objective responses (1 CR and 4 PR) were seen in patients with breast cancer, including 3 patients with triple-negative disease. All of the patients with triple-negative breast cancer (5 of 5 patients) had stable disease or better on this therapy. One patient with triple-negative breast cancer, previously treated with 7 lines of therapy, achieved a CR and remained on treatment for 9 months. PRs and stable disease were each seen in 2 additional patients with triple-negative breast cancer (4 total patients). Due to these encouraging results suggestive of disease specific activity, a phase II trial in triple-negative breast cancer is under development.

One patient with previously untreated chondrosarcoma had durable SD for 6 months and remained on treatment for 12 months until new splenic lesions were reported on imaging, but his bulky primary tumor sites remained under control. Study therapy was well tolerated without toxicity at the planned phase II dose. While one patient is a limited sample, stable disease for 6 months in chondrosarcoma for which there is no accepted first line standard therapy is a notable observation that deserves further assessment.

While no MTD was reached in cohort B, further dose escalation was not explored. Experience in cohort A demonstrated that pemetrexed doses in excess of 750 mg/m^2^ led to unacceptable levels of mucositis. DLTs and delayed toxicity of the continuous dosing combination made escalation of intermittent sorafenib doses beyond 400 mg orally twice daily impractical. In addition, 2 patients required dose modification of both agents at dose level B8.

Based on response and toxicity data, dose level B7 (pemetrexed 500 mg/m^2^ IV day 1, sorafenib 400 mg twice daily on days 1–5, every 14 days) is the RP2D of the combination. This dose level was well tolerated and below the highest tolerable dose of 750 mg/m^2^ of pemetrexed with intermittent sorafenib 400 mg orally twice daily. Fewer dose delays and no dose modifications were required at this dose level compared to dose level B8. The 500 mg/m^2^ dose of pemetrexed has been selected for phase 2 testing in breast cancer. Four of the 5 responding patients with breast cancer were treated at this dose during this study, and higher doses of pemetrexed have been studied previously in lung cancer and were not associated with improvements in response rate or median overall survival [23].

Lack of expression of the tumor suppressor gene, PTEN, in multiple solid and hematologic malignancies has been strongly associated with a reduced response to chemotherapy and to more rapid disease progression and death [24]. Our results suggest that reduced expression of PTEN may correlate with the biological and clinical responses of patients. We view these results as hypothesis generating at this time and plan to do a prospective analysis of PTEN levels and function as part of the successor phase II clinical study in triple negative breast cancer. The decreased expression of PTEN in responders is contrary to our a priori assumptions regarding tumor resistance with loss of this biomarker. The present studies did not determine, in those patients expressing high levels of PTEN protein who also exhibited stable disease, whether the phosphatase activity of the PTEN enzyme was abolished due to a point mutation or reduced due to PTEN hyper-phosphorylation. Additional laboratory based studies are presently defining PTEN functionality in all patients from this trial, and whether reduced PTEN function and tumor cell sensitivity in any of these patients correlated with higher basal activities of AKT, mTOR, p70 S6K, and ERK1/2 as was observed in our pre-clinical studies.

It was observed that 20 of 33 patients (61%) had stable disease or tumor regression as a best response. This supports further evaluation of this drug combination, especially with intermittent sorafenib dosing, which was well tolerated, possibly with the addition of other novel agents to explore enhancement of toxic autophagy as an antineoplastic approach. Our laboratory is presently developing 3-drug combinations using the combination of pemetrexed with sorafenib as a backbone^1^.

## MATERIALS AND METHODS

### Drug supply

Commercial stock of pemetrexed (Alimta, LY231514; NSC 698037; Eli Lilly and Company) was obtained and provided at no cost to patients by the Virginia Commonwealth University (VCU) Massey Cancer Center. Sorafenib (Nexavar, BAY 43-9006; NSC 724772; Bayer Healthcare Pharmaceuticals) was obtained by VCU Massey Cancer Center or stock was provided by Bayer and provided at no cost to participants. Both drugs were provided through the VCU Health System Investigational Drug Service, unless a patient's diagnosis met FDA-indications for either drug and third-party authorization could be obtained, in which case the agent was obtained commercially.

### Patient eligibility

Patients must have had a diagnosis of advanced solid tumor malignancy with no potential curative treatment. Any number of prior lines of therapy was allowed. Ineligible patients included those with uncontrolled brain metastases, contraindication to antiangiogenic agents, arterial thromboembolic or embolic events within the past 6 months, major cardiac dysfunction, systolic blood pressure greater than 160 mm Hg or diastolic pressure greater than 100 mm Hg despite optimal medical management, inability to interrupt aspirin or non-steroidal anti-inflammatory agents for a 5-day period, serious uncontrolled infection (Common Terminology Criteria for Adverse Events [CTCAE] v 4; grade > 2), or known or presumed intolerance to pemetrexed or sorafenib. Patients unwilling or unable to take folic acid, vitamin B12, or dexamethasone, or with an inability to swallow or suspected malabsorption were also ineligible. Prior treatment with pemetrexed or sorafenib was allowed.

Eligible patients had to meet the following criteria: at least 18 years of age; aspartate transaminase (AST) or alanine transaminase (ALT) less than or equal to 3 times the upper institutional limit of normal (ULN); total bilirubin of less than or equal to 1.5 times the upper institutional limit; creatinine clearance of at least 45 mL/min by standard Crockcroft-Gault equation; INR less than or equal to 1.5, unless due to anticoagulants; hemoglobin levels of at least 8.5 g/dL; total white count of at least 3.0 × 10^9^/L; an absolute neutrophil count of at least 1.5 × 10^9^/L; and a platelet count of at least 80 × 10^9^/L for cohort A and at least 100 × 10^9^/L for cohort B. Initially, an Eastern Cooperative Oncology Group (ECOG) performance status score of 2 or less was required. A protocol amendment effective for cohort B modified the ECOG performance status inclusion criteria to a score of 1 or less for cohort B and further defined eligibility criteria by excluding any patients with grade 2 or greater neuropathy, low serum B12/folate levels, or platelets of less than 100 × 10^2^/L. Prior chemotherapy toxicities were allowed as long as they were stable and did not interfere with study drug toxicity assessment. Patients were required to have measurable or evaluable disease by RECIST v1.1 [25].

### Treatment plan

This study was designed as a phase I, non-randomized, dose-escalation study to determine the maximum-tolerated dose (MTD) for the pemetrexed and sorafenib combination, where pemetrexed was administered as a 15-minute infusion every 14 days. Sorafenib in cohort A was given orally continuously, starting concurrent with the first dose of pemetrexed. Sorafenib doses ranged from 200 to 400 mg twice a day and pemetrexed doses ranged from 500 to 1,000 mg/m^2^. Following a protocol revision, cohort A was closed to further accrual, and cohort B, exploring intermittent dosing of sorafenib with each dose of pemetrexed, was opened. In cohort B, sorafenib was given orally on days 1–5, starting the morning of each pemetrexed dose. Sorafenib doses ranged from 200 to 400 mg twice a day and pemetrexed doses ranged from 500 to 750 mg/m^2^. Pemetrexed treatment was repeated every 14 days in both cohorts. Disease status was assessed approximately every 8 weeks. Patients experiencing a partial or complete response (PR or CR) or stable disease (SD) were allowed to continue treatment indefinitely at the investigator's discretion. Pharmacokinetics were not performed in this study given that pemetrexed is cleared through the kidneys and sorafenib is metabolized in the liver.

### Study design, definition of DLT, and identification of the MTD and recommended phase 2 dose

A novel dose escalation matrix in which one or both agents could be escalated or de-escalated based on observed toxicities was used to define dose levels for cohort A [26]. For cohort B, a standard 3 + 3 dose-escalation design was used, with dose level expansion of up to 6 evaluable patients if a DLT was noted. The MTD was defined as the highest dose level at which fewer than 2 of 6 patients experienced DLT. The recommended phase 2 dose (RP2D) may not exceed than the MTD, but could be set to less than the MTD if the pattern of toxicity and dose modifications suggest that a lower dose would be preferred for further development.

The first 28 days of treatment comprised the DLT-evaluation period. For both cohorts, DLT was defined as any toxicity of grade 3 or greater that was determined to be possibly, probably, or definitely related to study treatment except for the following: (i) nausea, vomiting, or diarrhea in the absence of adequate prophylaxis and/or responsive to medical management; (ii) fatigue responsive to medical management; (iii) grade 3 hypertension; (iv) asymptomatic laboratory abnormalities; (v) electrolyte abnormalities that, once corrected, could be maintained with oral repletion; and (vi) maculo-papular rash. For cohort B, any of the following grade 3 events were also excluded as DLT events: hand-foot syndrome, anemia, thrombocytopenia, neutropenia, leukopenia, lymphopenia, or febrile neutropenia.

### Toxicity evaluation

NCI CTCAE v 4 was used for reporting adverse events.

### Response evaluation

Tumor masses were evaluated for response according to RECIST v 1.1 [25].

### Archival tumor tissue for biomarker analysis

Archival tumor tissue was analyzed by immunohistochemistry (IHC) for PTEN, Beclin 1, and PDGFRb expression. Tumor tissues were deparaffinized and rehydrated through graded alcohols to water. Heat-induced antigen retrieval was done with sodium citrate buffer (10 μM, pH 6.0) for PTEN staining and with Tris/EDTA buffer (pH 9.0) for Beclin 1 and PDGFRβ staining. The slides for staining with anti-PTEN or anti-PDGFRβ antibodies were washed with TBS containing 0.025% Tween 20, and the slides for staining with anti-Beclin 1 antibody were washed with PBS containing 0.1% Tween 20. Before staining, the slides were incubated for 10 minutes with Dual Endogenous Enzyme Block, an endogenous peroxidase inhibitor (EnVision+ Dual Link System-HRP (DAB+); DakoCytomation, Denmark). The slides were then blocked with 10% normal goat serum for 2 hours at room temperature with the diluent for each antibody as specified by the manufacturer. After washing, the slides were stained overnight at 4°C with anti-PTEN (1:100, #9559, Cell Signaling Technology), anti-Beclin 1 (1:1,000, ab62472, Abcam), or anti-PDGFRb (1:100, ab5511, Abcam) antibody. Antibody staining was visualized using EnVision+ Dual Link System-HRP (DAB+) according to the manufacturer's protocol. The slides were read by a clinical pathologist who was blinded to the identity of the patient specimens.

### Statistical analysis

Demographics, adverse events, DLTs, dose levels, and clinical responses were summarized by basic descriptive statistics such as frequency, proportion, mean, median, and range. An ordinal regression method with cumulative logits link was used to determine if there was a statistically significant association (*p* ≤0.05) between biomarker expression levels (high vs. low/null expression) and the ordinal clinical response (complete response [CR], PR, SD, and progressive disease [PD]).

### Human investigation studies

Studies were performed after Institutional Review Board approval and in accordance with an assurance filed with and approved by the Department of Health and Human Services. Informed consent was obtained from each patient. The ClinicalTrials.gov trial registration ID is NCT01450384.
